# Surgical Correction of Ascending Aortic Aneurysm Without Coronary Dilatation After Kawasaki Disease in a 3-Year-Old Child

**DOI:** 10.21470/1678-9741-2020-0727

**Published:** 2022

**Authors:** Rômullo M Santos, Maria Raquel B Massoti, Monica Raquel Gonzalez Coronel, Frederico Faria Maia, Leonardo Augusto Miana, Marcelo Biscegli Jatene

**Affiliations:** 1 Department of Pediatric Cardiac Surgery, Instituto do Coração, São Paulo, São Paulo, Brazil.

**Keywords:** Aortic Aneurysm, Mucocutaneous Lymph Node Syndrome, Communicable Diseases, Precipitating Factors, Genetic Predisposition to Disease, Child

## Abstract

Kawasaki disease (KD) is an inflammatory condition that may affect genetically predisposed individuals in pediatric ages after infectious/environmental triggering. An infrequent finding associated with KD is ascending aortic aneurysm during or after the acute phase of the disease. In this Multimedia presentation, we describe a three-year-old girl submitted to surgical treatment.

**Table:** Abbreviations, acronyms & symbols

AAA	= Ascending aortic aneurysm
CT	= Computed tomography
KD	= Kawasaki disease
RCA	= Right coronary artery
TTE	= Transthoracic echocardiogram

## CASE PRESENTATION

Kawasaki disease (KD) is a clinical condition in which some genetically predisposed individuals in pediatric ages, after infectious triggering, may develop immunological and inflammatory response against vascular tissue^[1,2]^.

Arterial complications may be present, most commonly observed as coronary aneurysms, but other arteries can be affected in a lower incidence, like the ascending aortic aneurysm (AAA), a very rare manifestation^[2-5]^.

The surgical treatment of AAA in pediatric ages may represent a follow-up free from aortic tissue dissection or rupture in this population^[6,7]^.

Herein we present a case of a three-year-old patient submitted to surgical correction of AAA after incomplete KD manifestation. Consent for publication was granted by the patient’s mother.

At 20 months of age, the previously healthy patient presented with daily fever (39°C), prostration, and oral erythema. She was diagnosed with an incomplete form of KD and received a high immunoglobulin dose for four weeks.

Computed tomography (CT) scan revealed a significant saccular aneurysm (31 mm, score of + 9.0) in ascending aorta and dilatation of aortic root (21 mm), with no other arterial or coronary dilatation. Preoperative transthoracic echocardiogram (TTE) showed normal left ventricular function and no aortic valve insufficiency or aortic annulus dilatation (15 mm). The aortic root and ascending aorta diameters by the TTE were 24 mm and 34 mm, respectively.

The patient was then referred to our cardiac center for follow-up. Repeated CT scan revealed rapid expansion of aortic root and ascending aorta diameters (3 mm in nine months) with respectively 26 mm and 36 mm. Therefore, elective surgical treatment was planned.

## TECHNICAL DESCRIPTION

Surgery was performed after a median sternotomy and cardiopulmonary bypass with moderate hypothermia, aortic cross-clamping, and infusion of Custodiol® as cardioplegic solution.

After opening the aneurysm, a high takeoff of right coronary artery (RCA) was identified and isolated from the aneurismatic sac. Sinotubular junction presented only mild dilatation.

The aneurysm was resected, and we performed interposition of 26-mm dacron graft (JOTEC® FlowWeave Bioesal) proximally anastomosed above the sinotubular level and distally to the normal ascending aortic tissue.

RCA was detached and reimplanted in the dacron graft because of its high takeoff above sinotubular junction and its involvement in the aneurismatic tissue. Native aortic valve and aortic root were preserved (video). See the surgical procedure in the attached video.

**Video:**
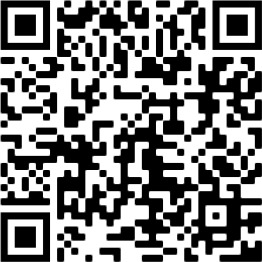
Surgical procedure.

The intraoperative transesophageal echocardiogram showed neither valvar dysfunction nor coronary blood flow obstruction. The patient presented uneventful postoperative recovery and was discharged from the intensive care unit on the postoperative 2^nd^ day and from the hospital on the postoperative 7^th^ day.

The postoperative histological findings were suggestive of previous vasculitis due to previous KD activity.

## COMMENT

Herein we present an infrequent manifestation of KD in early infancy: AAA without coronary involvement. In other previous series, peripheral artery aneurysms were associated with coronary aneurysms in all cases^[3,4]^.

It is not clear if early clinical treatment with high doses of intravenous immunoglobulin and oral aspirin is necessarily correlated with the prevention of aortic dilatation. However, these medications should be encouraged because of the observed benefit against coronary artery damage.

One hypothesis is that systemic arterial dilatation occurs even in properly clinically treated and asymptomatic individuals after KD^[3,5]^.

There are few reports about the surgical treatment of AAA after KD, and current guidelines do not state what the better approach is and when it should be surgically treated in the pediatric population^[6,7]^.

Nevertheless, in some previous series, aortic aneurysm surgical correction with native valve preservation in early childhood seems to be the technique of choice with good results in the late follow-up^[7,8]^.

## References

[1] Dietz SM, van Stijn D, Burgner D, Levin M, Kuipers IM, Hutten BA (2017). Dissecting kawasaki disease: a state-of-the-art
review. Eur J Pediatr.

[2] Cohen E, Sundel R (2016). Kawasaki disease at 50 years. JAMA Pediatr.

[3] Hoshino S, Tsuda E, Yamada O (2015). Characteristics and fate of systemic artery aneurysm after
kawasaki disease. J Pediatr.

[4] Zhao QM, Chu C, Wu L, Liang XC, Sun SN, He L (2019). Systemic artery aneurysms and kawasaki disease. Pediatrics.

[5] Ravekes WJ, Colan SD, Gauvreau K, Baker AL, Sundel RP, van der Velde ME (2001). Aortic root dilation in kawasaki disease. Am J Cardiol.

[6] Erbel R, Aboyans V, Boileau C, Bossone E, Bartolomeo RD, Eggebrecht H (2014). 2014 ESC guidelines on the diagnosis and treatment of aortic
diseases: document covering acute and chronic aortic diseases of the
thoracic and abdominal aorta of the adult. The task force for the diagnosis
and treatment of aortic diseases of the European society of cardiology
(ESC). Eur Heart J.

[7] Cattaneo SM, Bethea BT, Alejo DE, Spevak PJ, Clauss SB, Dietz HC (2004). Surgery for aortic root aneurysm in children: a 21-year
experience in 50 patients. Ann Thorac Surg.

[8] Moreau de Bellaing A, Pontailler M, Bajolle F, Gaudin R, Murtuza B, Haydar A (2020). Ascending aorta and aortic root replacement (with or without
valve sparing) in early childhood: surgical strategies and long-term
outcomes. Eur J Cardiothorac Surg.

